# Characterization of a Novel Porin-Like Protein, ExtI, from *Geobacter sulfurreducens* and Its Implication in the Reduction of Selenite and Tellurite

**DOI:** 10.3390/ijms19030809

**Published:** 2018-03-11

**Authors:** Mst. Ishrat Jahan, Ryuta Tobe, Hisaaki Mihara

**Affiliations:** Department of Biotechnology, College of Life Sciences, Ritsumeikan University, Kusatsu, Shiga 525-8577, Japan; ishrat.jahan014@gmail.com (M.I.J.); tober@fc.ritsumei.ac.jp (R.T.)

**Keywords:** porin, metalloid reduction, selenite, tellurite, *Geobacter sulfurreducens*

## Abstract

The *extI* gene in *Geobacter sulfurreducens* encodes a putative outer membrane channel porin, which resides within a cluster of *extHIJKLMNOPQS* genes. This cluster is highly conserved across the *Geobacteraceae* and includes multiple putative *c*-type cytochromes. In silico analyses of the ExtI sequence, together with Western blot analysis and proteinase protection assays, showed that it is an outer membrane protein. The expression level of ExtI did not respond to changes in osmolality and phosphate starvation. An *extI*-deficient mutant did not show any significant impact on fumarate or Fe(III) citrate reduction or sensitivity to β-lactam antibiotics, as compared with those of the wild-type strain. However, *extI* deficiency resulted in a decreased ability to reduce selenite and tellurite. Heme staining analysis revealed that *extI* deficiency affects certain heme-containing proteins in the outer and inner membranes, which may cause a decrease in the ability to reduce selenite and tellurite. Based on these observations, we discuss possible roles for ExtI in selenite and tellurite reduction in *G. sulfurreducens*.

## 1. Introduction

Both selenite and tellurite are highly soluble and exhibit high toxicity to organisms [1,2]. Bacteria can convert selenite and tellurite to less-toxic elemental selenium (Se^0^) and elemental tellurium (Te^0^), respectively [1,3,4]. These bacterial selenite/tellurite reductions have been shown to be environmentally and biotechnologically significant processes [1,2,4]. Selenite and tellurite reductions by bacteria are generally associated with the formation of nano-sized deposits of Se^0^ and Te^0^ either inside or outside the cell [2,3]. Selenite reduction can by catalyzed by a number of enzymes, such as periplasmic nitrite reductase [5], sulfite reductase [6], hydrogenase I [7], fumarate reductase [8], dimethyl sulfoxide reductase-like enzyme [9], and NAD(P)H-dependent flavoenzymes, including thioredoxin reductase and glutathione reductase [10,11], depending on bacterial species. Tellurite reduction can also be catalyzed by a variety of enzymes, including dihydrolipoamide dehydrogenase [12], NDH-II dehydrogenase [13], nitrate reductase [14], glutathione reductase [15], catalase [16], and by the poorly characterized flavin- [17] and NAD(P)H-dependent [18] reductases. The involvement of reduced thiols, such as glutathione, in selenite/tellurite reduction has been suggested to take place in the cytosol of cells [19,20]. Recent studies have uncovered yet another side to selenite and tellurite metabolism: these chalcogen oxyanions are able to serve as electron acceptors in the respiratory chain and support the anaerobic growth of certain bacteria [21,22].

*Geobacter sulfurreducens*, a gram-negative anaerobic bacterium, belongs to a class of microorganisms that use a wide range of respiratory electron acceptors, from an organic compound, fumarate, to insoluble metal oxides such as manganese and iron oxides, for anaerobic growth [23]. The transfer of electrons to the extracellular substrate requires one of two inner membrane *c*-type cytochromes (Cyt*c*s), ImcH and CbcL, depending on the reduction potential of the extracellular acceptor [24], and the PpcA-type periplasmic triheme *c*-type Cyt*c*s [25]. A conductive porin-cytochrome (Pcc) complex consisting of the OmaB multiheme Cyt*c*, OmbB porin-like protein, and OmcB multiheme lipoprotein Cyt*c* is involved in electron transfer outside the cell to extracellular electron acceptors like Fe(III)-oxides [26,27,28]. The *G. sulfurreducens* genome contains at least five other putative *pcc* gene clusters [29], among which *extABCD* (*gsu2645-2642*) is implicated as an electrode specific electron conduit [30]. In addition to these, a combination of conductive type IV pili (PilA) and multiheme Cyt*c*s, such as OmcS, OmcE, OmcZ, and PgcA, are implicated in electron transfer beyond the outer membrane [27]. Meanwhile, the soluble electron acceptors fumarate and Fe(III) citrate, are presumed to be reduced by the inner membrane-bound fumarate reductase (FrdCAB) [31] and by the putative Pcc OmaB, OmbB, and OmcB [26], respectively.

Soluble toxic inorganic species, such as uranyl ion, arsenate, and selenite, also serve as electron acceptors for *G. sulfurreducens*. The reduction of soluble U(VI), As(V), and Se(IV) to insoluble U(IV), As(III), and Se^0^, respectively, prevents them from leaching into groundwater, offering the potential for bioremediation of contaminated soils, sediments, industrial effluents, and agricultural drainage waters [32,33]. The majority of studies of anaerobic respiration by *G. sulfurreducens* have focused on the mechanism of electron transfer to transition metals, radionuclides, and electrodes, while the mechanism of electron transfer to metalloids, such as selenium and tellurium compounds, remains poorly understood [28,30]. Selenite reduction by *G. sulfurreducens* results in the formation of an insoluble red precipitate of amorphous Se^0^ on the surface of the cells, as the predominant end product [33]. The red amorphous Se^0^ is stable after several months, suggesting a role for membrane components of the *G. sulfurreducens* cells, in stabilizing the Se^0^ nanospheres [33]. A 15 kDa Cyt*c* was identified as a protein bound to biogenic extracellular Se^0^ nanospheres produced during the growth of the bacterium in the presence of selenite [33]. These observations suggest that selenite reduction occurs at the surface or the periplasmic space of the cell. Se^0^ formation is also implicated in a pivotal component of selenite reduction in another metal-reducing bacterium, *S. oneidensis* [34]. However, reduction of tellurite by *G. sulfurreducens* has not yet been reported.

The outer membrane of gram-negative bacteria plays pivotal roles in bacterial survival in a wide range of environments, serving as a protective barrier and allowing for the uptake of nutrients [35,36]. Outer membrane proteins are major components of the outer membrane and include anchoring lipoproteins and transmembrane β-barrel proteins, such as porins, substrate-specific transporters, and active transporters [36]. The transmembrane β-barrel proteins are characterized by the number of anti-parallel β-strands, ranging in number from eight to twenty-four [37]. Porins are the most abundant and important transmembrane β-barrel proteins of the outer membrane, comprising up to 2% of the entire protein content of the cell [35]. They serve as water-filled open channels allowing the passive penetration of hydrophilic molecules, which are discriminated depending on their overall physiochemical properties, such as size, hydrophobicity, and charge [38]. Canonical porins, such as *Escherichia coli* OmpC and OmpF, have broad specificity and allow a range of molecules into the cell [39], while *E. coli* PhoE and *Pseudomonas aeruginosa* OprP and OprO, which have specific binding sites for certain molecules [40,41], are examples of substrate-specific porins. General porins are also permeated by antibiotics and are therefore associated with bacterial mechanisms of antibiotic resistance [42]. Moreover, recent studies have shown that porins are implicated in respiration [43], electron transfer [44], and pathogenesis [45].

The OmpJ porin, the most abundant outer membrane protein in *G. sulfurreducens*, has been shown to be required for respiration of soluble Fe(III) citrate, as well as insoluble Fe(III) oxide and Mn(IV) oxides [44]. However, the effect of OmpJ in extracellular electron transfer may be indirect, as OmpJ is proposed to be required for the integrity of the periplasmic space necessary for folding and functioning of periplasmic and outer membrane electron transport components. Similarly, one of the two major porins of *S. oneidensis* MR-1, OmpS38 (also termed Omp35), is indirectly involved in respiration and/or growth on non-oxygen electron acceptors, such as Fe(III) citrate, fumarate, and nitrate [43,46]. Expression of OmpS38 does not respond to changes in osmolality, and OmpS38 appears to be unimportant in the diffusion of β-lactam antibiotics in *S. oneidensis* [46]. However, a certain outer membrane porin-like protein forms a trans-outer membrane Pcc complex, together with an outer membrane and periplasmic Cyt*c*, to transfer electrons from the periplasmic space and across the outer membrane to the extracellular electron acceptors [26,27,28,29]. Such porin-like proteins include the well-characterized *S. oneidensis* MtrB [47], as well as OmbB and OmbC of *G. sulfurreducens* [28]. In *S. oneidensis* MR-1, each of the *mtrC-mtrA-mtrB* and *mtrD-mtrE-mtrF* gene clusters encode a periplasmic 10-heme Cyt*c* (MtrA/MtrD), a porin-like outer membrane protein with 28 predicted trans-outer membrane motifs (MtrB/MtrE), and an outer membrane 10-heme Cyt*c* (MtrC/MtrF) [29,47]. The *ombB-omaB-omcB* and *ombC-omaC-omcC* gene clusters in *G. sulfurreducens* PCA are analogous to the two *mtr* gene clusters of *S. oneidensis* MR-1, although they share no identity at the amino-acid sequence level, with exception of the heme-binding motifs of the Cyt*c*s [27]. Insights are needed into other porins that could have roles in selenite/tellurite (metalloid) reduction.

The *G. sulfurreducens* genome contains more than 100 genes predicted to encode Cyt*c*s, based on the presence of the CXXCH heme *c* binding motif [48]. Previous proteome analyses showed that most of the putative Cyt*c*s are detectable in the extract of *G. sulfurreducens* [49,50]. However, only a limited number of Cyt*c*s have been characterized in detail [26,27,29,51]. We have previously found in silico that the translational product of *gsu2937*-*gsu2936* may be a novel Cyt*c*-like protein containing a selenocysteine residue [52]. The putative selenoprotein is encoded by a unique gene cluster (*gsu2937*-*gsu2930*, designated *extKLMNOPQS*) that is highly conserved in *Geobacter* species [53]. Conserved Cyt*c*s in this gene cluster were predicted to be 4-heme (GSU2930, ExtS), 10-heme (GSU2934, ExtN), 12-heme (GSU2935, ExtM), and 5-heme (GSU2937-2936, ExtKL) proteins. The gene cluster also contains an inner-membrane-bound *b*-type cytochrome (GSU2932, ExtP) and Rieske Fe-S protein (GSU2933, ExtO), suggesting an electron transport function for this cluster. The *extKLMNOPQS* cluster could be further extended to include *extH* (*gsu2940*), *extI* (*gsu2939*), and *extJ* (*gsu2938*), which code for a putative rhodanese-like protein, a putative porin-like protein, and a small protein (104 amino-acids) of unknown function. The *extHIJKLMNOPQS* gene cluster is not analogous to known *pcc* gene clusters [29], and ExtI shows a weak similarity to phosphate-selective porins [40,54], suggesting the possibility that the gene cluster may be involved in the penetration of a certain molecule or an as-yet unidentified electron transfer, either at the surface or in the periplasmic space of the *G. sulfurreducens* cell. In this study, we have made efforts to functionally characterize the putative porin-like protein ExtI of *G*. *sulfurreducens* PCA. ExtI did not notably respond to changes in osmolality and was not required for transport of β-lactam antibiotics into the periplasm. Further investigation suggested several possible roles for ExtI in selenite/tellurite reduction, such that it may be a selenite/tellurite channel porin and/or indirectly involved in the reduction of selenite and tellurite by anchoring certain cytochrome proteins that are required for this process, to the periplasmic space, outer membrane, and inner membrane.

## 2. Results

### 2.1. Conservation of ExtI Homologs in Other Geobacteraceae

**Figure 1:**
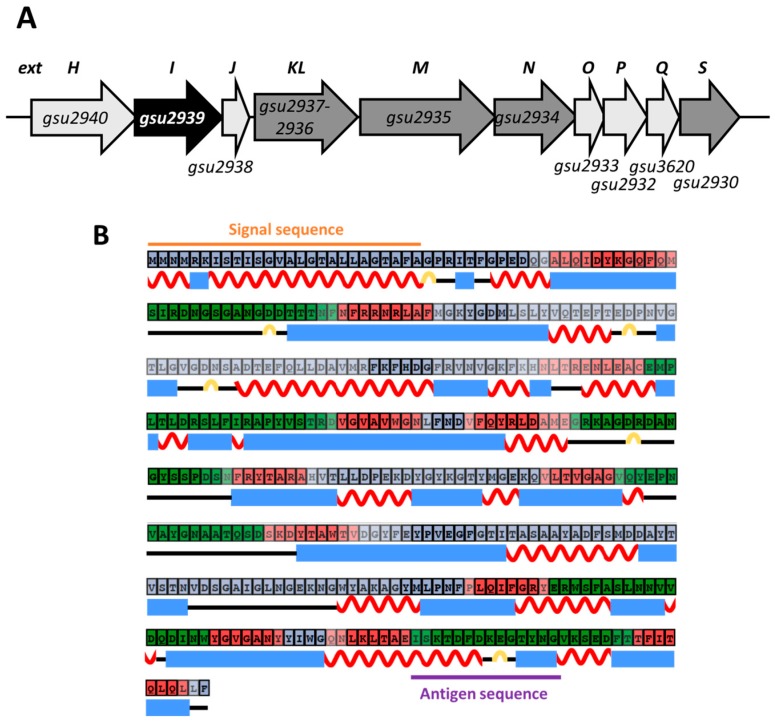
Bioinformatics analysis of ExtI. (**A**) The *extHIJKLMNOPQS* gene cluster in *G. sulfurreducens*. The black arrow indicates the gene for a porin-like protein, *extI* (*gsu2939*), and the dark grey arrows indicate the genes for putative Cyt*c* proteins, *extKL* (*gsu2937-2936*), *extM* (*gsu2935*), *extN* (*gsu2934*), and *extS* (*gsu2930*). The light gray arrows indicate the genes for a rhodanese-like protein, *extH* (*gsu2940*); a small protein, *extJ* (*gsu2938*); a Rieske iron-sulfur protein, *extO* (*gsu2933*); a cytochrome *b*, *extP* (*gsu2932*); and a membrane protein, *extQ* (*gsu3620*). (**B**) Prediction of the secondary structure of the ExtI protein. The amino-acid sequence of ExtI was computationally analyzed, and the secondary structure and the signal region were predicted. The *N*-terminal signal sequence (1–26 a.a.) predicted by SignalP 4.1 and the transmembrane α-helix region in the signal sequence (7–26 a.a.) predicted by TMHMM are depicted. Amino-acids in red, green, and gray boxes are predicted to be transmembrane, extracellular side, and periplasmic side, respectively. Red wavy lines, blue squares, and yellow lines represent α-helices, β-strands, and turns, respectively. The purple line shows the peptide sequence of the antigen used for preparation of the anti-ExtI antibody.

The *extI* gene is located in an operon-like gene cluster containing 10 genes ranging from *extH* to *extS* (Figure 1A). The gene cluster is highly conserved in the genomes of at least the ten other members of the *Geobacteraceae* for which complete sequences are available at GenomeNet (available online: http://www.genome.jp/): *G. sulfurreducens* KN400, *G. bemidjiensis*, *G. daltonii* FRC-32, *G. uraniireducens*, *G. metallireducens*, *G. lovleyi*, *G. anodireducens*, *Geobacter* sp. M21, *Geobacter* sp. M18, and *Geoalkalibacter subterraneus*. The translational product of *extKL* was proposed to be a Cyt*c* protein (49.5 kDa) containing a selenocysteine residue [52]. Other gene products for the gene cluster are predicted as follows: ExtH, a rhodanese-like protein (49.0 kDa); ExtI, a putative porin (45.2 kDa); ExtJ, a hypothetical protein (10.6 kDa); ExtM, a Cyt*c* with 12 hemes (68.3 kDa); ExtN, a Cyt*c* with 10 hemes (47.3 kDa); ExtO, a Rieske iron-sulfur protein (14.1 kDa); ExtP, a cytochrome *b* (23.2 kDa); ExtQ, a membrane protein (16.8 kDa); and ExtS, a lipoprotein Cyt*c* (28.8 kDa) [53,55].

Close homologs of ExtI showing more than 64% sequence identity were found only in the genomes of the *Geobacteraceae* family, while its homologs with moderate (35–60%) identity were identified in species such as *Desulfurispirillum indicum*, *Deferribacter desulfuricans*, *Denitrovibrio acetiphilus*, *Desulfurivibrio alkaliphilus*, *Thiohalobacter thiocyanaticus*, *Thioalkalivibrio sulfidiphilus*, and *Desulfocapsa sulfexigens*. Among these, only *D. indicum* has been known to reduce selenite [56]. However, none of these ExtI homologs have been studied. Although ExtI is annotated as a phosphate-selective porin in sequence databases, it showed little (about 12%) sequence identity with hitherto reported phosphate-selective porins, OprP and OprO of *Pseudomonas aeruginosa* [40] or PhoE of *E. coli* [41]. Among other porins so far characterized, *Methylophilus methylotrophus* FmdC, which is involved in the transport of short-chain amides and urea [57], exhibited the highest sequence identity (19%) with ExtI. In addition, neither the Pcc complex-forming porins (OmbB, OmbC, and MtrB) nor Fe(III)-reduction-associated porins (OmpJ and OmpS38) from the metal-reducing bacteria *G. sulfurreducens* and *S. oneidensis* showed significant sequence homology to ExtI. These results suggest that ExtI is a novel type of porin-like protein that is unique to the *Geobacteraceae* family.

### 2.2. Membrane Localization of ExtI

In bacteria, the structure of the outer membrane porin family is generally characterized by an even number of β-strands; usually 14, 16, or 18 strands [36]. The result of the secondary structure prediction algorithm CFSSP [58] showed that ExtI had more β-strands than α-helices. The transmembrane detection and topology prediction program BetAware [59] suggested that ExtI contained 12 transmembrane segments constituting a β-barrel structure (Figure 1B). A SignalP 4.1 server (available online: http://www.cbs.dtu.dk/services/SignalP/) [60] prediction showed that ExtI had an *N*-terminal signal sequence (amino-acids 1–26), with a predicted cleavage site between amino-acids Ala26 and Gly27 (orange line in Figure 1B). In addition, a TMHMM analysis [61,62] indicated that a transmembrane helix (Ile7-Ala26) was present in the predicted *N*-terminal signal sequence, which may be required for membrane translocation [63]. Consistently, the PSORTb program [64] led to the prediction of a subcellular localization for ExtI to the outer membrane. Furthermore, ExtI has only one cysteine residue and a predicted low isoelectric point (theoretical pI of 4.76) which are known characteristics of outer membrane proteins of gram-negative bacteria [38]. These results suggested that ExtI may be a porin-like β-barrel protein, likely to be localized in the outer membrane. After cleavage of the signal peptide, a mature protein was predicted to consist of 380 amino-acid residues with a molecular mass of 42.7 kDa.

**Figure 2:**
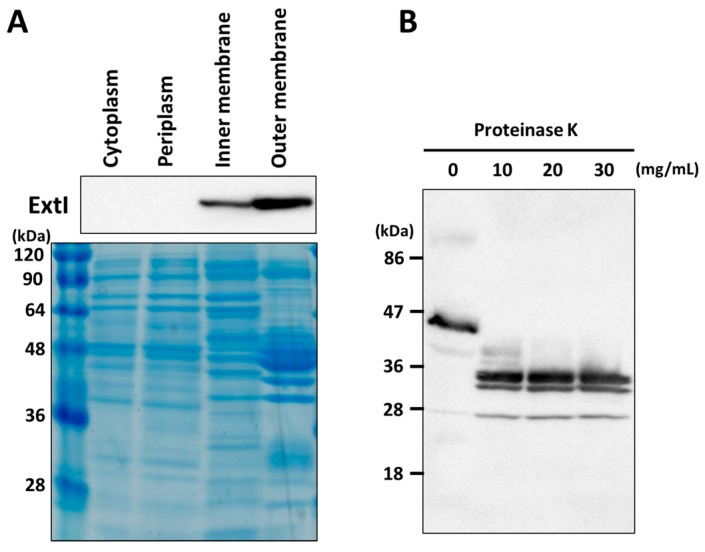
Cellular localization of ExtI. (**A**) Subcellular fractionation and Western blotting. Each cellular fraction was isolated, and the localization of ExtI was analyzed by Western blotting (upper panel). A Coomassie-stained gel is shown as the loading control (lower panel). The numbers at the left indicates the sizes of the marker proteins in kDa; (**B**) the effects of proteinase K treatment on ExtI integrity. Cells were treated with different concentrations (0 to 30 mg/mL) of proteinase K to analyze the surface exposure of ExtI, and the degraded products were analyzed by Western blotting.

To examine the intracellular localization of ExtI in *G. sulfurreducens*, cell fractionation analysis was performed using a specific anti-ExtI antibody, raised against a synthetic peptide corresponding to part of the predicted extracellular region (Ile356-Gly389) of ExtI (Figure 1B). Western blot analysis of the subcellular fractions showed that the majority of the immunoreactive 43-kDa protein, corresponding to the mature ExtI, was detected in the outer membrane fraction, but not in the cytosol or periplasm fractions (Figure 2A). A certain amount of ExtI was also detected in the inner membrane fraction, most likely due to contamination with the outer membrane fraction. Nevertheless, the CBB-stained SDS-PAGE gel showed significantly different protein patterns between the outer and inner membrane fractions, indicating a fair separation of the two fractions. In fact, abundant protein bands were observed around 45 kDa on the CBB-stained gel, consistent with the presence of the major porin OmpJ in the outer membrane of *G. sulfurreducens* [44]. Therefore, possible interaction of ExtI, even if directly or indirectly, with a certain periplasmic and/or cytoplasmic membrane protein(s) could not be ruled out at this stage.

The outer membrane localization of ExtI was further examined by an in situ proteinase K treatment experiment of intact cells of *G. sulfurreducens* as described for bacterial surface proteins [65]. The proteinase K treatment of whole cells leads to the digestion of the exposed region of outer membrane proteins, while subsurface regions remain protected. No complete digestion of ExtI was observed after the treatment, even with increased concentrations up to 30 mg/mL of proteinase K (Figure 2B), although the PeptideCutter program [66] predicted 206 proteinase K cutting sites in the ExtI sequence. In contrast, severe degradation of the total protein, dependent on the increased amount of proteinase K, was observed on a CBB-staining gel (data not shown). The proteinase K treatment resulted in the complete loss of mature ExtI and the appearance of three immunoreactive protein fragments, with molecular masses of 34, 32, and 28 kDa, suggesting that only limited regions of ExtI were highly accessible to proteinase K. Taken together, these data greatly supported our assertion that ExtI is located and largely embedded in the outer membrane, as expected of a porin-like protein.

### 2.3. ExtI Production Does Not Respond to Changes in Osmolality or Phosphate Concentration

**Figure 3:**
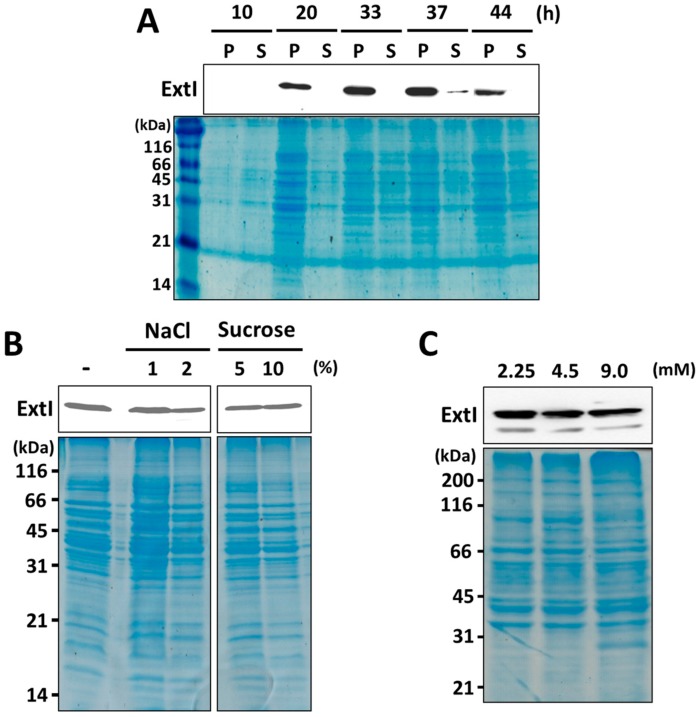
Analysis of ExtI protein expression in *G. sulfurreducens*. (**A**) The growth-dependent expression levels of ExtI. Cells were cultured in an NBAFYE medium and analyzed by Western blotting to examine the relative expression levels of ExtI at various growth points. The pellet and supernatant from the disrupted cells are referred to as P and S, respectively. A Coomassie-stained gel is shown as the loading control. The numbers at the left indicates the sizes of the marker proteins in kDa; (**B**) the effects of osmolality on ExtI expression. Cells were incubated in either NBAFYE medium (−) or the same medium containing NaCl or sucrose at the indicated concentrations for two days. Membrane proteins from the cells were extracted, and the ExtI expression levels were analyzed by Western blotting. A Coomassie-stained gel is shown as the loading control; (**C**) the effects of phosphate concentration on ExtI expression levels. Cells were cultured in FWA medium at different concentrations of phosphate (2.25, 4.5, and 9 mM), and the expression levels of ExtI in the membrane fractions were analyzed by Western blotting. A Coomassie-stained gel is shown as the loading control.

To investigate whether the expression levels of ExtI change during cell growth, we carried out a Western blot analysis of cells grown in an NBAFYE medium containing 15 mM acetate and 40 mM fumarate. As shown in Figure 3A, the expression levels of ExtI in the early stationary phase (33 and 37 h) were slightly higher than those in the mid-log phase (20 h). The expression levels of ExtI then declined significantly by 44 h. This result suggested that ExtI is constitutively expressed with a slight increase at the early stationary phase in an NBAFYE medium. Next, we examined production of ExtI under various degrees of osmolality, as several “classical” porins are known to be involved in bacterial adaptation and response to changes in osmolality of the environment [67]. The insoluble membrane fractions of the cells grown in the presence or absence of NaCl or sucrose were analyzed by Western blot. As shown in Figure 3B, no significant difference in the levels of ExtI was observed under conditions of high osmolality (1–2% NaCl and 5–10% sucrose), suggesting that ExtI does not respond to changes in osmolality in a significant manner. Under more severe osmotic stress conditions (5% NaCl and 20% sucrose), *G. sulfurreducens* failed to grow well. Further, we examined whether production of ExtI responds to phosphate concentrations, because ExtI is categorized within a family of phosphate-selective porin O and P, in the Pfam protein family database [68], whose expression levels are induced under phosphate starvation, as reported for *P. aeruginosa* OprO [69] and *E. coli* PhoE [70]. The *G. sulfurreducens* cells were grown under different concentrations of phosphate (2.25–9.0 mM), and levels of ExtI in the membrane fractions were analyzed by Western blot. No significant difference was observed in the expression levels of ExtI in the cells grown under phosphate-limited (2.25 mM) conditions (Figure 3C). These results suggested that ExtI is constitutively expressed and may not be regulated by a well-known osmotic response two-component regulatory system, such as the EnvZ-OmpR system and the PhoB-PhoR system, as seen for the transcriptional control of several classical channel porins [67,71,72].

### 2.4. Construction of the ExtI-Deficient Strain

Our data described above, together with the occurrence of the *extI* gene in a limited group of bacteria, suggested that ExtI might play a key role in the physiology of those metal-reducing bacteria. To elucidate the physiological role of ExtI, an *extI*-deficient (∆*extI*) strain was generated by gene replacement with a kanamycin-resistance gene (*Km^r^*) (Figure S1A). The accurate replacement of the chromosomal *extI* gene by the *Km^r^*-cassette was confirmed by the amplification of a 2.2-kbp fragment from the genomic DNA of ∆*extI* (Figure S1B) and by DNA sequencing of the PCR product. Western blot analysis further confirmed the absence of ExtI in the outer membrane fraction of the ∆*extI* strain. RT-PCR analysis of the mRNAs of the downstream genes, *extKL*, *extM*, and *extN*, showed no significant differences between the wild-type and ∆*extI* strains in the transcript levels of the genes, suggesting that the insertion of the *Km^r^*-cassette did not cause a polar effect on downstream genes (Figure S1C). Western blot analysis using an anti-ExtKL antibody also exhibited no influence on the ExtKL protein levels. These data clearly demonstrate successful disruption of the *extI* gene without affecting expression of the downstream genes in the *extHIJKLMNOPQS* gene cluster in ∆*extI*.

### 2.5. ExtI Appears Unimportant for Fumarate Respiration, Permeation of β-Lactam Antibiotics and Fe(III) Reduction

The ∆*extI* strain grew well, comparable to the wild-type strain, in either NBAFYE medium containing 15 mM acetate and 40 mM fumarate or FWA minimum medium containing 20 mM fumarate as the electron acceptor (Figure S2). This suggests that ExtI has no essential role in fumarate respiration or the permeation of nutrients under the conditions examined. Mutants of the neoclassical major porin genes, *G. sulfurreducens ompJ* and *S. oneidensis ompS38*, are known to exhibit impaired anaerobic reduction of Fe(III) citrate [43,44,46]. We found that the ∆*extI* strain showed no significant differences in the reduction of Fe(III) citrate in comparison with the wild-type strain. Although not tested in this study, the previous study shows that the deletion of *extHIJKL* has no effect on the ability of *G. sulfurreducens* to reduce insoluble Fe(III) oxide [30]. Since permeation by β-lactam antibiotics is a common characteristic of nonspecific channel porins, loss of major classic porins generally causes a decreased susceptibility to a certain β-lactam antibiotics [73]. To examine whether ExtI plays a significant role as a nonspecific channel, we assessed the sensitivity of the ∆*extI* strain to two different β-lactam antibiotics, ampicillin and penicillin. Loss of ExtI appeared not to affect the susceptibility to penicillin and ampicillin, as compared with the wild-type strain (Figure S3), suggesting that ExtI may not be a major nonspecific channel porin. Taken together, these results suggested that ExtI is neither a canonical nonspecific porin, a general major porin required for Fe(III) citrate, nor a component of an insoluble Fe(III)-reducing Pcc complex.

### 2.6. ExtI Deficiency Affects the Conversion of Selenite/Tellurite to Se^0^/Te^0^

**Figure 4:**
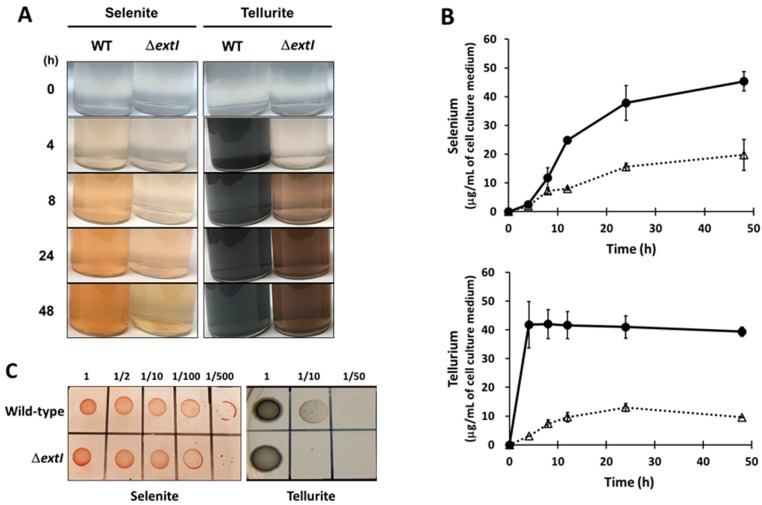
The effects of *extI* deficiency on selenite and tellurite reduction. (**A**) The colorimetric change observed during selenite and tellurite reduction. Wild-type (WT) and *extI*-deficient mutant (∆*extI*) cells were anaerobically cultured in FWA medium containing 500 µM selenite (left panels) or 500 µM tellurite (right panels) as only the electron acceptors; (**B**) wild-type (circle and solid line) and ∆*extI* (triangle and dotted line) cells were anaerobically cultured in FWA medium containing 500 µM selenite (upper panel) or 500 µM tellurite (lower panel) as the sole electron acceptor. The amounts of selenium and tellurium precipitated were measured by HG-AFS. Experiments were conducted independently two times and error bars represent standard deviation; (**C**) selenite and tellurite susceptibility. The wild-type and ∆*extI* cells were anaerobically cultured in NBAFYE medium. After adjusting for bacterial OD, cells were diluted as indicated with saline and spotted onto the NBAFYE agar plate with 500 µM selenite (upper panel) and 500 µM tellurite (lower panel), and then cultured for 3 days.

The data presented above and the location of *extI* within the *extHIJKLMNOPQS* gene cluster including putative Cyt*c* genes, suggested that ExtI may be associated with an electron transfer system for a molecule other than fumarate and Fe(III). In accordance with this hypothesis, we noticed in our preliminary studies that the disruption of Cyt*c* genes downstream of *extI* caused a phenotype of *G. sulfurreducens* showing an impaired ability to reduce selenite to red Se^0^, in a medium containing selenite. Therefore, we examined the effects of ExtI deficiency on the ability of the bacterium to reduce selenite and its cognate compound tellurite, in the FWA medium containing selenite or tellurite as the sole electron acceptor. When cells were grown in the selenite-containing medium, the ∆*extI* strain produced markedly decreased amounts of red precipitates of Se^0^, as compared to the wild-type strain (Figure 4A). Similarly, the formation of black precipitates of Te^0^, in the culture of the ∆*extI* strain grown in the tellurite-containing medium, was significantly impaired in comparison with the wild-type strain (Figure 4A). A quantitative analysis of Se^0^ and Te^0^ precipitates formed in the cultures, was carried out using hydride generation atomic fluorescence spectrometry (HG-AFS). The ∆*extI* strain showed significantly lower rates of production of Se^0^ and Te^0^, as compared to the wild-type strain (Figure 4B). The amounts of Se^0^ and Te^0^ produced by ∆*extI* were decreased to 30% and 25%, respectively, compared with those produced by the wild-type strain at 48 h cultivation. We found that the precipitates formed in the liquid culture of ∆*extI* with tellurite, showed a brownish-black color in contrast to the black precipitates of Te^0^ produced by the wild-type strain, suggesting the production of a different form of Te^0^ nanostructure in these strains.

To further assess the role of ExtI in the transformation of selenite/tellurite to Se^0^/Te^0^, we examined the growth ability of the cells on NBAFYE agar medium containing selenite or tellurite. We found that the ∆*extI* strain displayed slightly decreased growth rates in the presence of selenite and tellurite, as represented by the faint colonies of the ∆*extI* mutant at higher dilution factors (1/500 for selenite and 1/10 for tellurite) as compared with the wild-type strain (Figure 4C). These data suggest that the loss of ExtI impedes the conversion of selenite and tellurite to Se^0^ and Te^0^, respectively. One possible explanation for this is that ExtI is not a channel porin for selenite/tellurite, as the inefficient permeation of the chalcogen oxyanions across outer membranes into periplasm/cytoplasm should confer resistance to these toxic oxyanions in the ∆*extI* mutant strain. However, we prefer an alternative hypothesis that ExtI may function as a selenite-selective channel porin (see Section 3).

### 2.7. ExtI Is Important for the Localization or Stabilization of Certain Heme-Containing Proteins

**Figure 5:**
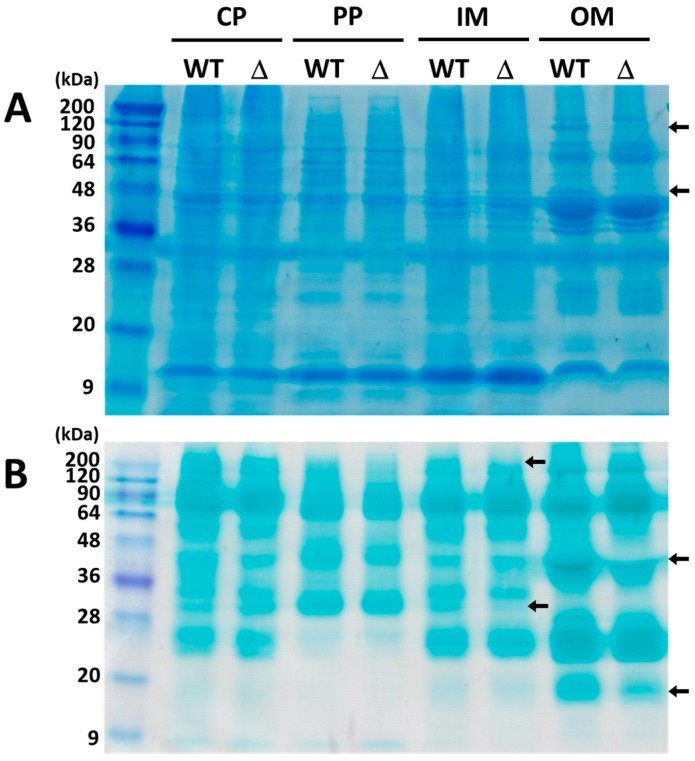
Comparison of the total protein pattern in cell fractionations and the heme staining. (**A**) Total protein pattern of the subcellular fractions. Each subcellular fraction was isolated from the wild-type (WT) and the *extI* deficient mutant (Δ) strains, and the fraction proteins (20 μg) were separated by SDS-PAGE and stained with CBB; (**B**) heme-staining. Proteins of each subcellular fraction were separated by SDS-PAGE, and heme-containing proteins were stained with *N*,*N*,*N*′,*N*′-tetramethylbenzidine. Arrows indicate representative changed proteins between wild-type and *extI* deficient mutant. CP, cytoplasm; PP, periplasm; IM, inner membrane; OM, outer membrane.

Based on the amino-acid sequence, ExtI was predicted not to have a catalytic function in directly reducing selenite and tellurite, since it has no binding motif for electron-transferring cofactors, such as flavin, hemes, or iron-sulfur clusters. This raised the possibility that ExtI may be a component of an electron transfer complex required for selenite/tellurite reduction, in a manner somewhat analogous to the Pcc complex [27,29,55]. Another intriguing hypothesis is that ExtI may affect the reduction of selenite/tellurite by playing a key role in the activation, maturation, or stabilization of the component of an as yet unknown selenite/tellurite-reducing protein. The organization of *extI* in the Cyt*c*-rich *extHIJKLMNOPQS* gene cluster seems to be in accordance with both of the above notions. To address these issues, we analyzed the compositions of total proteins and heme proteins, in the subcellular fractions of the wild-type and *∆extI* strains. The CBB-stained gel showed that there were few, if any, differences between the wild-type and the mutant strains, in the protein compositions of the cytosolic, periplasmic, and inner membrane fractions (Figure 5A). Only a few CBB-stained proteins and heme-stained proteins were significantly affected by the loss of ExtI in the outer membrane fractions. The most markedly affected proteins in the outer membranes were those at 45 and 120 kDa, which were diminished in ∆*extI* in the CBB-stained gal (Figure 5A). In the heme-stained gel, 15- and 45-kDa proteins in the outer membrane were significantly decreased in the mutant, as compared with those in the wild-type strains (Figure 5B). In addition, 30- and 200-kDa heme proteins in the inner membrane were also affected by the mutation. However, the changes in the protein compositions, induced by the loss of ExtI, was markedly milder than those observed for the OmpJ-deficient strain, in which the integrity of the cell surface is also affected [44]. The results suggested that ExtI has only a minimal role in the integrity of the cell surface structure. Instead, ExtI may be important to maintain the integrity of a certain protein(s), such as heme-containing cytochrome(s) involved in selenite/tellurite reduction. Nevertheless, the possibility that ExtI may serve as a selective channel porin to selenite/tellurite cannot be ruled out at present (see Section 3).

## 3. Discussion

### 3.1. Can a Similar Mechanism Be Employed between Phosphate Channels and Selenite/Tellurite Channels?

ExtI is annotated in the genome of *G. sulfurreducens* as a gene that codes for a putative phosphate-selective porin family protein, and exhibits weak (<13%) sequence similarity with the well-studied phosphate-selective porins OprO and OprP of *P. aeruginosa* [54,74]. The OprP crystal structure reveals structural features that are important for the anion selectivity of the channel, i.e., the so-called arginine ladder, including eight Arg residues that extend from the extracellular side of the pore, and a lysine cluster including nine Lys residues on the periplasmic side of the channel [75]. Two central phosphate-binding sites are formed by residues D94, Y62, S124, S125, K121, K126, R34, and R133. Consequently, both OprP and OprO are rich in Arg and Lys residues (47 residues in total per subunit). In contrast, ExtI contains 16 Arg and 16 Lys residues (32 residues in total), and only a few substrate-binding residues (D94 and K121 in OprP) are conserved in ExtI (Figure S4). In addition, the previous study showed that genes in the *pst*-*pho* operon, which is associated with a high-affinity phosphate uptake system, are significantly increased under phosphate-limiting conditions in *G. sulfurreducens* [76]. In contrast, there was no significant change in the levels of ExtI, even under phosphate-limited conditions (Figure 3). Moreover, a slightly higher (<20%) sequence identity is found between ExtI and FmdC, which is suggested to be involved in the transport of short-chain amides and urea [57]. Therefore, it is unlikely that ExtI functions as a phosphate-selective porin in *G. sulfurreducens*. The Δ*extI* mutant showed a decreased ability to transform selenite/tellurite to Se^0^/Te^0^, as compared with the wild-type strain (Figure 4), suggesting a role for ExtI in selenite/tellurite uptake into the periplasmic space. In connection with this, the existence of a similar uptake mechanism for selenite/tellurite and phosphate has been demonstrated. In *E. coli*, tellurite uptake through cytoplasmic membranes into the cytosol can be mediated by the PitA phosphate transporter [77]. A major role of the phosphate transporter Pho90 in selenite uptake, has been suggested in *Saccharomyces cerevisiae* [78]. In addition, a phosphate transporter, OsPT2, is involved in selenite uptake in rice [79]. Other mechanisms are also known, such as the monocarboxylate transporter, Jen1p in *S. cerevisiae*, for selenite uptake [80] and the acetate transporter, ActP in *E. coli*, for tellurite uptake [77]. Nevertheless, no previous reports exist that implicate an outer membrane channel porin in the uptake of selenite or tellurite.

### 3.2. The Association of ExtI with Other Uncharacterized Genes Is Highly Conserved in Geobacteraceae

The most notable feature of ExtI is the coexistence of its gene in a single cluster, together with the genes coding for a putative rhodanese, multiple Cyt*c*s, and homologs of the cytochrome *bc* complex (Figure 1). The high conservation of this cluster in *Geobacter* species suggests that the *extHIJKLMNOPQS* cluster is physiologically significant. A previous comparative genomics study of six *Geobacter* genomes, indicated that the conservation of Cyt*c*s is poor, while an abundance of Cyt*c*s (an average of 79) are found in all *Geobacter* species [53]. Most of the Cyt*c*s required in *G. sulfurreducens* for growth on extracellular acceptors were not conserved in all species, including OmcE, OmcF, OmcS, OmcT, OmcX, OmcZ, and MacA. However, there were only 64 conserved Cyt*c*s among a total of 471 Cyt*c*s identified in the six *Geobacter* genomes. These 64 conserved Cyt*c*s form nine protein families. Intriguingly, the four of the nine families that are conserved in all species are ExtKL, ExtM, ExtN, and ExtS, which are encoded together in a ExtI-encoding gene cluster in each genome. An inner-membrane-bound *b*-type cytochrome (ExtP) and Riske Fe-S protein (ExtO) within the gene cluster, are homologous to the core of the cytochrome *bc* complexes (complex III), which catalyzes a key step in electron transport, providing the electrical link between the inner membrane and periplasm [81]. However, the protein that provides this link in the *Geobacteraceae* has not been characterized, making the well-conserved *extHIJKLMNOPQS* cluster a promising candidate for further analysis. The relation between ExtI and the multiple Cyt*c*s in this highly-conserved cluster warrants further investigation.

### 3.3. Possible Role of ExtI

Our preliminary study suggested that *extKL* encodes a Cyt*c*-like selenoprotein, which is involved in selenite reduction. The data presented in this paper showed that the mutation in *extI* had no effects on the transcription of its downstream genes, including *extKL* (Figure S1C). Therefore, the defect in selenite/tellurite reduction caused by the loss of ExtI is not due to polar effects on downstream genes, including *extKL*. Although some of the outer and inner membrane proteins, including heme-containing proteins, are affected by the loss of ExtI (Figure 5), no apparent general perturbation was found in the cell, as demonstrated by the growth of the mutant in the presence of fumarate (Figure S2) and in the Fe(III) citrate reduction. These results suggest that ExtI does not play a general structural role in keeping the integrity of cell surface, but rather plays a specific role in a process associated with selenite/tellurite reduction. What is the link between selenite/tellurite reduction and the role of ExtI? One possible explanation for the decreased ability for selenite/tellurite reduction in Δ*extI* is that ExtI interacts with some proteins involved in selenite/tellurite reduction and/or Se^0^/Te^0^ formation, possibly including ExtKL or other electron transfer proteins encoded by the *extHIJKLMNOPQS* gene cluster, to support their integrity or localization in outer membrane, periplasmic space, or inner membrane.

*G. sulfurreducens* is able to produce Se^0^ nanoparticles on the surface of the cell [33]. Studies of other bacterial species suggest that certain proteins, such as the outer membrane protein OmpC [82] and other porins [83], are involved in the formation of Se^0^ nanoparticles. In the formation of Se^0^ in *T. selenatis*, a novel ~95 kDa protein, SefA, has been identified as a Se^0^-forming protein [84]. SefA is proposed to provide reaction sites for Se^0^ nanosphere creation or prevent subsequent Se^0^ aggregation. In *G. sulfurreducens*, a 15-kDa Cyt*c* was identified as an Se^0^-bound protein, which is suggested to be involved in the formation of extracellular Se^0^ nanoparticles [83]. Our results show that a 15-kDa heme-containing protein, which is possibly identical to the Se^0^-bound 15-kDa Cyt*c*, was diminished in the Δ*extI* strain (Figure 5). This raises the possibility that the observed phenotype of decreased selenite/tellurite conversion to Se^0^/Te^0^ in Δ*extI* may be associated with the loss of the 15-kDa cytochrome, which may be important for extracellular Se^0^/Te^0^ formation. If this is the case, the association of Se^0^/Te^0^-forming Cyt*c* with a potential selenite/tellurite uptake channel, may provide an important clue as to the mechanism of transformation of selenite/tellurite to extracellular Se^0^/Te^0^.

Our data show that the Δ*extI* strain was more sensitive than the wild-type strain to the presence of selenite/tellurite in the solid agar medium containing fumarate (Figure 4C). Mutation or inhibition of a multidrug efflux pump generally increases the drug susceptibility of bacteria, as reported for the antibiotic-sensitive TolC mutants [85] and the OmpW mutant [86]. This raises the possibility that the increased selenite/tellurite-sensitivity in Δ*extI* may be related to a potential role for ExtI in the direct efflux of these toxic chalcogen oxyanions. However, detoxification of selenite/tellurite generally depends on oxyanion reduction, followed by Se^0^/Te^0^ nanoparticle formation on the surface of cells [2,4] as also seen in *G. sulfurreducens* [33]. Therefore, a role for ExtI in direct selenite/tellurite efflux without forming Se^0^/Te^0^ nanoparticle is unlikely to explain our data. On the other hand, if the selenite/tellurite reduction is able to benefit the growth of *G. sulfurreducens*, even in the presence of fumarate, then the loss of ability to reduce selenite/tellurite would cause a retarded growth phenotype on a medium containing selenite/tellurite. In this line of reasoning, our data is also suggestive of a role of ExtI in selenite/tellurite uptake and/or utilization. Selenite reduction and subsequent selenide transfer by a selenium delivery protein to selenophosphate synthetase, is proposed to be an important process in selenoprotein biosynthesis [87,88,89]. However, the link between selenite reduction and selenophosphate synthesis remains unclear. Biosynthesis of selenoprotein requires a source of selenium, usually in the form of selenite in bacteria [88]. The *G. sulfurreducens* genome contains at least 8 selenoprotein genes [90]. Therefore, selenite import through ExtI into the periplasmic space might be important for the subsequent uptake of a fraction of selenite into the cytoplasm, to achieve the biosynthesis of a selenoprotein that is involved in selenite reduction. Obviously, ExtKL is a candidate for such a selenoprotein required for selenite reduction.

### 3.4. The Association between Selenite Reduction and Iron Reduction

Previous study shows that ExtKL was significantly more abundant during growth with Fe(III) versus growth with fumarate as the electron acceptor [50]. The cell fractionation and proteomic identification also showed that ExtKL and ExtS are abundant in the outer and cytoplasmic membranes, whereas ExtM was detected in outer and cytoplasmic membranes as well as periplasm [50]. Another proteome study showed that ExtN and ExtQ are more abundant during growth with Fe(III) oxides versus Fe(III) citrate [49]. In addition, ExtH and ExtKL (and presumably ExtI also) are shown to be directly regulated by the Fe(III) uptake regulator, Fur. All these reports demonstrated an upregulation of the *extHIJKLMNOPQS* gene cluster in response to Fe(III), especially Fe(III) oxide. This seems to suggest a possible link between the role of the *extHIJKLMNOPQS* gene cluster and the uptake/utilization of iron. However, there was no requirement of ExtI for Fe(III) citrate reduction. It has also been previously reported that *extHIJKL* is not required for electron transfer to Fe(III) oxide and low-potential electrodes [30]. On the other hand, an intriguing link is suggested between Fe(III) reduction and the biogeochemical cycle of selenium [91]. In sulfur-rich marine environments, anaerobic bacterial communities are thought to play a key role in the formation of FeS, pyrite (FeS_2_), and S^0^ [92]. In such environments, selenium levels are known to correlate positively with the content of pyrite, chlorite, and illite [91]. Thus, regulation of the selenium metabolic pathway, together with Fe(III) reduction in response to Fe(III), could be of significance in the selenium geochemical cycle.

## 4. Materials and Methods

### 4.1. Chemicals, Bacterial Strains and Growth Conditions

All chemicals were purchased from Wako Pure Chemical Industries (Osaka, Japan) and Nacalai Tesque (Kyoto, Japan). *G. sulfurreducens* PCA was obtained from Leibniz Institute DSMZ-German Collection of Microorganisms and Cell Cultures (Braunschweig, Germany). *G. sulfurreducens* cells were cultured under strict anaerobic conditions at 30 °C. The NBAFYE medium contained an NB trace mineral solution and vitamin solution [93] supplemented with 15 mM acetate, 40 mM fumarate, 0.1% yeast extract and 1 mM cysteine. The freshwater acetate (FWA) medium [93] contained 20 mM acetate as the electron donor and either 20 mM Fe(III) citrate or 40 mM fumarate as the electron acceptor.

### 4.2. Subcellular Fractionation

Subcellular fractions of *G. sulfurreducens* were prepared according to an established method [51] with some modifications. Briefly, *G. sulfurreducens* cells were grown to mid-log to stationary growth phases under anaerobic conditions in NBAFYE medium. The harvested cells were washed twice with PBS buffer and resuspended in PBS buffer containing 350 mM sucrose. Following another centrifugation, cells were re-suspended in 10 mL of 250 mM Tris-HCl buffer (pH 7.5) and allowed to stand at room temperature for 10 min with 10 μL of 0.5 M EDTA (pH 8.0). Sucrose (70 mM) was added, and the mixture was incubated at room temperature for 20 min, followed by the addition of 15 mg/mL lysozyme and incubation for 30 min at 30 °C. The mixture was centrifuged (20,000× *g*, 10 min, 4 °C), and the supernatant was used as the periplasmic fraction. The pellet was resuspended in 100 mM Tris-HCl buffer (pH 7.5) containing DNase I, and the spheroplasts were disrupted by sonication on ice. The crude extract was centrifuged, and the pellet was reserved as the cell debris fraction. The supernatant was centrifuged for 30 min at 20,000× *g* at 4 °C, and the supernatant was used as the cytoplasmic fraction. The pellet was re-suspended with 100 mM Tris-HCl buffer (pH 7.5). Following the addition of an equal volume of 100 mM Tris-HCl (pH 7.5) containing 2% (*w*/*v*) lauroylsarcosine, the suspension was incubated for 15 min at room temperature and then centrifuged for 30 min at 125,000× *g* at 4 °C. The supernatant was reserved as the inner membrane fraction, and the pellet was reserved as the outer membrane fraction.

### 4.3. Protein Quantification

Protein concentrations were determined in two ways as follows. (1) The Bradford method with bovine serum albumin as a standard, using the Quick Start Bradford Dye Reagent (Bio-Rad, Hercules, CA, USA) for soluble protein quantification. (2) The 2D-quant kit (GE Healthcare, Little Chalfont, UK) for insoluble protein quantification, in which concentrations were determined according to the manufacturer’s instructions after the proteins were treated with 2% SDS to solubilize them.

### 4.4. SDS-PAGE and Western Blotting

SDS-PAGE analyses were performed using 12% polyacrylamide gels. Proteins were stained with Coomassie Brilliant Blue R-250. Western blotting was performed using polyvinylidene difluoride membranes (Invitrogen, Carlsbad, CA, USA) and an iBlot Dry Blotting System (Invitrogen, Carlsbad, CA, USA), according to the manufacturer’s instructions. For ExtI detection, a polyclonal antibody raised against a synthetic peptide (ISKTDFDKEGTYNG) corresponding to a partial sequence of ExtI was used (Eurofins Genomics, Tokyo, Japan). Immunoreactive proteins were detected using the Super Signal West Femto Maximum Sensitivity Substrate reagent (Thermo Fisher Scientific, Waltham, MA, USA) and a Lumino image analyzer LAS-4000 (Fujifilm, Tokyo, Japan).

### 4.5. In Situ Proteinase K Treatment

Surface exposure of the ExtI protein was examined with proteinase K according to a previously described method [94] with modification. Cells were grown in NBAFYE medium and harvested at the mid-exponential phase. After two washes with 10 mM Tris-HCl buffer (pH 8.0) containing 10 mM MgCl_2_ and 2% NaCl, cells were resuspended with the same buffer, to a final concentration of 63 mg of wet cells per mL. Samples were treated with various concentrations (0 to 30 mg/mL) of proteinase K for 5 min at room temperature, and a protease inhibitor cocktail was added to stop the proteolytic reaction. The cells were harvested and washed twice with the same buffer containing the protease inhibitor cocktail. The cell suspensions (10 mg protein/mL) were diluted 1:2 in SDS-sample buffer and boiled for 10 min and analyzed by SDS-PAGE and Western blotting.

### 4.6. Susceptibility Assays with Antibiotic and Chalcogen Oxyanions

The susceptibility assays were carried out as previously described [51]. Briefly, cells in mid-log phase were adjusted between the wild-type and the *extI-*deficient strains with fresh NBAFYE medium, followed by 10-fold serial dilutions. A 10 μL aliquot of each dilution was spotted onto NBAFYE agar plates containing 1.25 μg/mL of each antibiotic, or 500 μM of selenite or tellurite. The plates were anaerobically incubated at 30 °C for five days or longer before being read. For each strain, the analysis was performed in triplicate and repeated at least three times independently.

### 4.7. Gene Disruption

A DNA fragment for the deletion mutant of the *extI* gene was constructed by crossover PCR, replacing the +618 to +982 coding regions with a kanamycin resistance gene (*Km^r^*). Briefly, the upstream region of *extI* in the *G. sulfurreducens* genome was amplified with the primers Up-Fwd and Up-Rev, and the downstream region of *extI* with the primers Down-Fwd and Down-Rev. The *Km^r^* fragment was PCR-amplified with primers *Km^r^*-Fwd and *Km^r^*-Rev. The primer sequences are shown in Table S1. The three PCR-amplified fragments were used as templates in a PCR reaction, using the primers Up-Fwd and Down-Rev to amplify a 2.2-kbp DNA fragment. Preparation of electrocompetent cells and transformation of *G. sulfurreducens* have been described previously [93]. Electroporation was performed using the gene transfer equipment, GTE-10 (Shimadzu, Kyoto, Japan).

### 4.8. RT-PCR Analysis

Total RNA extraction from *G. sulfurreducens* strains was performed using a Sephasol-RNA I Super G kit (Nacalai Tesque, Kyoto, Japan) according to the manufacturer’s instructions. The synthesis of cDNA was performed using the ReverTra RT-PCR master mix with a gDNA remover kit (Toyobo, Osaka, Japan) according to the manufacturer’s instructions. Primer sequences are shown in Table S1. PCR was performed using a Thermal cycler MJ Mini (Bio-Rad Laboratories, Hercules, CA, USA) according to the instructions of a KOD Plus Neo PCR kit (Toyobo, Osaka, Japan). All samples were run in duplicate including a negative control.

### 4.9. Heme-Staining

Detection of heme-containing proteins on 12% SDS-PAGE gels was carried out as previously described [95]. For electrophoresis, β-mercaptoethanol was omitted in the sample buffer, and boiling time was reduced to 3 min.

### 4.10. Determination of Se^0^, Te^0^ and Fe(II)

Se^0^ and Te^0^ formed in *G. sulfurreducens* culture containing selenite and tellurite, respectively, were corrected by centrifugation, washed with PBS twice, and resuspended in 1 M phosphate buffer (pH 7.0). Aqua regia (nitric acid/hydrochloric acid = 1/3 (*v*/*v*)) was added to the precipitate suspensions, mixed well, and neutralized with sodium hydroxide. Selenium and tellurium in the samples were determined by HG-AFS (Millennium Excalibur, PSA, Orpington, UK) as described previously [96]. The analysis conditions were as follows: injection volume, 100 μL; acid carrier, 50% (*v*/*v*) HCl; and reductant, 0.7% (*w*/*v*) NaBH_4_ in 0.1 M NaOH. The Selenium-PS Analytical Lamp (P849SF PHOTRON) and Tellurium-PS Analytical Lamp (P855SF PHOTRON) were used for measuring selenium and tellurium concentrations, respectively. The concentrations of Fe(II) were spectrophotometrically quantified using a 0.1% ferrozine reagent at 562 nm as previously described [97]. Standard curves were prepared using Fe(II) ethylenediammonium sulfate tetrahydrate.

### 4.11. Bioinformatic Analysis

Protein sequences were obtained at the KEGG database (available online: http://www.kegg.jp/kegg/). Protein sequence similarity searches, multiple sequence alignment, and phylogenetic analyses were performed using the BLAST and TREE programs in the GenomeNet database (available online: http://www.genome.jp/). TMHMM (available online: http://www.cbs.dtu.dk/services/TMHMM/) [61,62] was used for transmembrane region prediction, BetAware (available online: https://betaware.biocomp.unibo.it/BetAware/) [59] was used for transmembrane β-barrel detection, and CFSSP (available online: http://www.biogem.org/tool/chou-fasman/) [58] was used for secondary structure prediction. The PSORTb algorithm (http://us. expasy.org) [64] and SignalP-3.0 (available online: http://www.cbs.dtu.dk/services/SignalP-3.0/) [63] were used to predict the cellular localization of the ExtI protein. Proteinase K cutting sites were identified using PeptideCutter (available online: http://web.expasy.org/peptide_cutter) [66].

## 5. Conclusions

The present study demonstrated that ExtI is an outer membrane porin-like protein. ExtI neither responded to changes in osmolality or phosphate starvation, nor was permeable to β-lactam antibiotics. The Δ*extI* strain showed a decreased ability to transform selenite and tellurite to Se^0^ and Te^0^, respectively, while the mutation had no significant impact on fumarate or Fe(III) citrate reduction. The loss of ExtI mildly affected the amount of certain heme-containing proteins in outer and inner membranes. Based on the predicted function of the genes in the same gene cluster, together with the available knowledge from omics-type studies in the literature, possible roles for ExtI in selenite/tellurite reduction are discussed, including those as a selenite/tellurite channel porin and as an anchor protein for selenite/tellurite-reducing proteins. Future studies are underway to investigate the detailed function of ExtI, as well as proteins encoded by the *extHIJKLMNOPQS* gene cluster.

## Supplementary Materials

Supplementary materials can be found at http://www.mdpi.com/1422-0067/19/3/809/s1.

## Abbreviations

Cyt*c*	Cytochrome *c*
Pcc	Porin-cytochrome
HG-AFS	hydride generation atomic fluorescence spectrometry
