# Supplementary material for: Characterization of a Novel Porin-Like Protein, ExtI, from Geobacter sulfurreducens and Its Implication in the Reduction of Selenite and Tellurite
Source: Int J Mol Sci. 2018 Mar 11;19(3):809. doi: 10.3390/ijms19030809 (PMC5877670; doi:10.3390/ijms19030809)
Supplement: Supplementary file 1 [file ijms-19-00809-s001.pdf]

Supplementary Materials

Supplementary Table S1. Primers used in this study

Number	Primer name	Sequence (5´ – 3´)
#1	Up-Fwd	ACCATCAGCACCACGATCTTGCCCAGCGG
#2	Up-Rev	CTGGTATGCCAAGGCCGGTTACATGCTCCC
#3	Down-Fwd	ACTCCGAGGGTACCCACGTTGGGATCTTCG
#4	Down-Rev	TCGTCGCCTTTCACCTCGACAATGGTCGGC
#5	Km^r-Fwd	CGAAGATCCCAACGTGGGTACCCTCGGAGTGGATGAATGTCAGC TAC
#6	Km^r-Rev	GGGAGCATGTAACCGGCCTTGGCATACCAGAGAAGGCGGCGGT GGAATCG
#7	Del_check_Fwd	CTTGACGGCATCGCCTACTACAACTCCAAC
#8	Del_check_Rev	CGATGCAGGAGCTGGAATCATACATCTTTG
#9	GSU2939_F	TCGATCCCGAGAAGGATTAC
#10	GSU2939_R	TGTCCACATTGGTGGATACG
#11	GSU2937_F	GAGAAAAACGGCTGGTATGC
#12	GSU2937_R	TGATGAACGTGGTGAAGTCC
#13	16S RNA_F	TGAGACACGGTCCAGACTCCTAC
#14	16S RNA_R	TCATTTCTTCCCTCCCGACA
#15	GSU2935_F	AATGCTACGGCTGTCATACGAAATA
#16	GSU2935_R	TTTCCCCTTGAAGGTAGAGACGTAG
#17	GSU2934_F	CCAGTTCATCCTTTACCATTCGGATTT
#18	GSU2934_R	GGTCCCAGAAGGAATCGAGAGAAAG

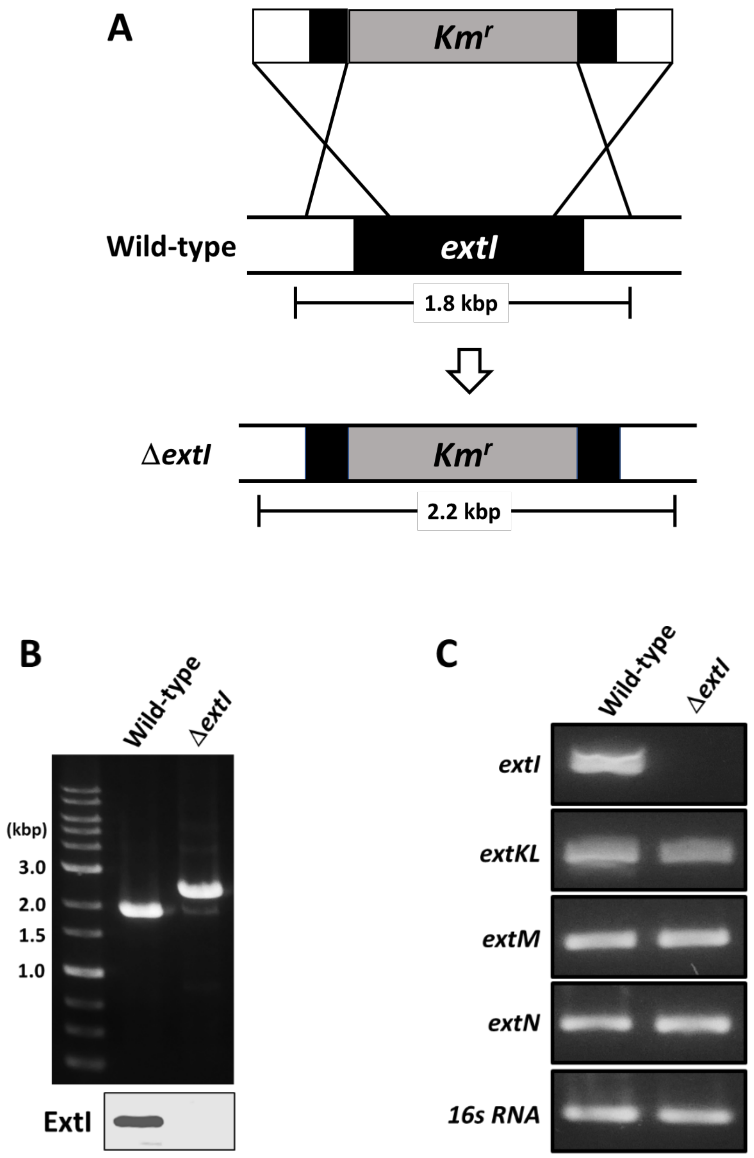

Supplementary Figure S1. Construction of an *extI*-deletion mutant (*ΔextI*). (**A**) Homologous recombination scheme for gene disruption. The *extI* gene was disrupted with a kanamycin resistance gene (Km^r) in wild-type *G. sulfurreducens* as described in the *Materials and Methods*. (**B**) The knockout mutation in the genome was confirmed by PCR (upper panel) using the genomic DNA and primers #7 and #8 shown in Supplementary Table S1 and Western blotting (lower panel) using an ExtI-specific antibody. (**C**) The mRNA levels of *extI*, *extKL*, *extM*, *extN*, and *16S rRNA* in the wild-type and the *ΔextI* strains were analyzed by semi-quantitative RT-PCR using each primer set shown in Supplementary Table S1.

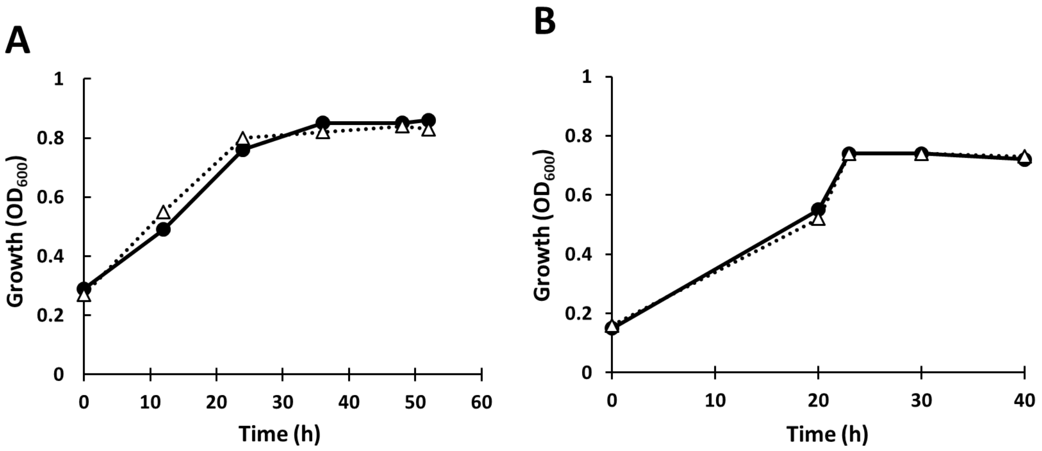

Supplementary Figure S2. Effect of *extI* deficiency on cell growth. Wild-type (circle and solid line) and Δ*extI* (triangle and dotted line) were anaerobically cultured in (**A**) NBAFYE medium containing 40 mM fumarate and (**B**) FWA medium containing 20 mM fumarate. Growth was measured by determining the optical density at 600 nm (OD_{600}).

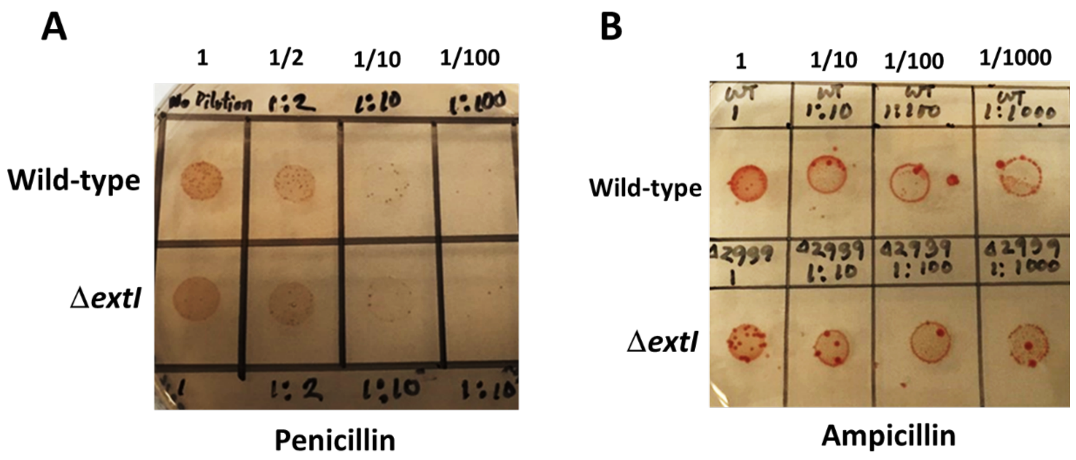

Supplementary Figure S3. Antibiotic susceptibility and permeability assay. Wild-type and Δ*extI* were anaerobically cultured in NBAFYE medium, and cells were diluted with saline and spotted onto the NBAFYE agar plate containing 1.25 µg/mL of (**A**) penicillin and (**B**) ampicillin and then cultured for 3 days.

```
                                           1*
PaeOprP     MIRRHSCKGV GSSVAWSLLG LAISAQSLAG TVTTDGADIV IKTKGGLEVA TTDKEFSFKL    60
PaeOprO     MIRKHSLG-- ---FVASALA LAVSAQAFAG TVTTDGADIV IKTKGGLEVA TTDKEFSFKL    55
ExtI        MMNMRKIS-- ------TISG VALGTALLAG TAFAG--PRI TFGPEDQGAL QIDYKGQFQM    50
            *:. :.           :  . :*:.:  :** *. :.    :      .  .    * : .*::

PaeOprP     GGRLQADYGR FDGYYTNNGN TADAAYFRRA YLEFGGTAYR DWKYQINYDL SRNVGNDSAG   120
PaeOprO     GGRLQADYSR FDGFYTKNGN TADAAYFRRA FIELGGTAYK DWKYQINFDL SHNTGSSDNG   115
ExtI        SIRDNGSGAN GDDTTTNFN- ------FRRN RLAFMGKYGD MLSLYVQTEF TEDPNVGTLG   103
            . * :.. ..  *.  *: .        ***   : : *.     .  :: ::: . : . .  *
              94*                             121*
PaeOprP     YFDEASVTYT GFNPVNLKFG RFY-TDFGLE KATSSKWVTA LERNLTYDIA DWVNDNVG-T   178
PaeOprO     YFDEASVTYT GFNPVNLKFG RFD-PDFGLE KATSSKWVTA PERNAAYELA DWINTHQDGM   174
ExtI        VGDNSADTEF QLLDAVMRFK FHDGFRVNVG KFKHNLTREN LEACEMPLTL DRSLFIRAPY   163
              *::: *    :  . ::*   .    ..:  * . .       *         *

PaeOprP     GIQASSVVGG MAFLSGSVFS ENNNDTDGDS VKRYNLRGVF APLHEPGNVV HLGLQYAYRD   238
PaeOprO     GAQVNSTLAD MAYLSAGVSA KDADDSDGDS VKQFNFRGVF APMHEAGNVL HVGVNYAYRD   234
ExtI        VSTRDVGVAV WGNLFNDVFQ YRLDAMEGRK AGDRDANGYS SPDS---NFR YT--ARAHVT   218
                .  :.   . *  .*      :  :* . .   : .*   :*     *.  :     *:

PaeOprP     LEDSAVDTRI RPRMGMRGVS TNGGNDAGSN GNRGLFGGSS -AVEGLWKDD SVWGLEGAWA   297
PaeOprO     LDDTAFDSRI RPRLGMRGIA TSGGNDAGDN GNRATFGGVS NSPAGSYKDD SVWGLEGAWA   294
ExtI        LLDPEKDYGY KGTYMGEKQV LTVGAGVQYE PNVAYGNAAT ---QSDSKDY TAWTVDGYFE   275
            * *.  *    :     .     . * ..  :  * .  .. :     .  ** : .* ::* :

PaeOprP     LGAFSAQAEY LRRTVKAER- DREDLKASGY YAQLAYTLTG EPRLYKLDGA KFDTIKPENK   356
PaeOprO     MGPFSAQAEY LARKLKADDN AYKDIKAKGY YAQLAYTLTG ESRQYKLEGA KFDSVKPENK   354
ExtI        Y-PVEGFGTI TASAAYADFS MDDAYTVSTN VDSGAIGLNG EKNGWYAKAG YMLPNFP---   331
              .... .         *:     .  ...     . *  *.* * . :  ...  : .  *

PaeOprP     EIGAWELFYR YDSIKVEDDN IVVDSATREV GDAKGKTHTL GVNWYANEAV KVSANYVKAK   416
PaeOprO     EIGAWEVFYR YDNIKVEDDN VVADTATREV GDTKAKAHNL GVNWYVNDAV KISAAYVKAK   414
ExtI        ----LQIFGR YERWSFASLN NVVDQDINWY GVGAN----- YYIWGQNLKL TAEISKTDFD   382
                 ::* * *:  .. . *  *.*   .   *             *  *  : . .   .. .

PaeOprP     TDKISNANGD DSGDGLVMRL QYVF                                          440
PaeOprO     TDKITNNNGD DDGDGFVTRL QYVF                                          438
ExtI        KEGTYNGVKS EDFTTFITQL QLLF                                          406
            .:   *  .  :.  : : :* * :*
```

Supplementary Figure S4. Comparison of the amino acid sequence of ExtI with those of the phosphate-selective porins. The multiple alignment of *G. sulfurreducens* ExtI, *P. aeruginosa* OprP, and *P. aeruginosa* OprO was constructed using a ClustalW program. The first amino acid (G, 1*) of the mature proteins, which are assumed to be formed after cleavage of the potential signal peptides, are highlighted in black, and the conserved D (94*) and K (121*) are highlighted in green and blue, respectively. Amino acid sequences were obtained from the KEGG databases (PaeOprP, PA3279; PaeOprO, PA3280).
